# Are researchers moving away from animal models as a result of poor clinical translation in the field of stroke? An analysis of opinion papers

**DOI:** 10.1136/bmjos-2019-100041

**Published:** 2020-02-24

**Authors:** Pandora Pound, Rebecca Ram

**Affiliations:** Safer Medicines Trust, Kingsbridge, UK

**Keywords:** stroke, animal models, neuroscience, opinion

## Abstract

**Objectives:**

Despite decades of research using animals to develop pharmaceutical treatments for patients who have had a stroke, few therapeutic options exist. The vast majority of interventions successful in preclinical animal studies have turned out to have no efficacy in humans or to be harmful to humans. In view of this, we explore whether there is evidence of a move away from animal models in this field.

**Methods:**

We used an innovative methodology, the analysis of opinion papers. Although we took a systematic approach to literature searching and data extraction, this is not a systematic review because the study involves the synthesis of opinions, not research evidence. Data were extracted from retrieved papers in chronological order and analysed qualitatively and descriptively.

**Results:**

Eighty eligible papers, published between 1979 and 2018, were identified. Most authors were from academic departments of neurology, neuroscience or stroke research. Authors agreed that translational stroke research was in crisis. They held diverse views about the causes of this crisis, most of which did not fundamentally challenge the use of animal models. Some, however, attributed the translational crisis to animal–human species differences and one to a lack of human in vitro models. Most of the proposed solutions involved fine-tuning animal models, but authors disagreed about whether such modifications would improve translation. A minority suggested using human in vitro methods alongside animal models. One proposed focusing only on human in vitro methods.

**Conclusion:**

Despite recognising that animal models have been unsuccessful in the field of stroke, most researchers exhibited a strong resistance to relinquishing them. Nevertheless, there is an emerging challenge to the use of animal models, in the form of human-focused in vitro approaches. For the sake of stroke patients there is an urgent need to revitalise translational stroke research and explore the evidence for these new approaches.

## Introduction

Despite decades of research using animals to develop pharmaceutical treatments for patients who have had a stroke, few therapeutic options exist. The vast majority of interventions successful in preclinical animal studies have turned out to either have no efficacy in humans, as with NXY-059^1^ or calcium channel blockers,^2^ or to be harmful to humans, as with diaspirin,^3^ emlimobab,^4^ selfotel^5^ and tirilazad,^6^ all of which increased the risk of death when taken to clinical trials. The failed quest for a neuroprotective agent is infamous; of more than 1000 candidate neuroprotective drugs tested in animals, not one was found to benefit humans with stroke.^7^

Currently, the only effective options available to those with haemorrhagic stroke consist of controlling blood pressure and admission to a stroke unit. For ischaemic stroke, the main options include admission to a stroke unit, secondary prevention using antiplatelets (eg, aspirin), and recanalisation, either pharmaceutically with thrombolytics or mechanically with endovascular thrombectomy. Although more treatment options are available for ischaemic stroke, thrombolytics can only be given to selected patients who present to a centre of expertise within 4.5 hours post stroke. (Clinical trials of thrombolytics for stroke followed on from their success with heart attack; animal studies were conducted to establish dosing^8^ but did not play a direct role in clinical translation.^9^) Currently, 11%–12% of patients receive thrombolysis in England, Wales and Northern Ireland,^10^ but around half of those who receive the treatment remain dependent or die.^11^ Similarly, only about 10% of patients who have a stroke are estimated to be eligible for endovascular thrombectomy.^12^ Against this background, stroke remains the second leading cause of death worldwide and the second most common cause of disability-adjusted life years.^13^ Despite the decline in death rates and stroke incidence in most regions, stroke is still prevalent and disabling, and population growth and ageing may result in a greater absolute pool of people at risk of stroke.^13^ Clearly, there is still a pressing need to develop effective responses to stroke that will benefit more than a minority of patients.

Given the unmet need of stroke patients, and in view of translational disappointments, we explore whether there is evidence of a move away from animal models in the field of translational stroke research. Kuhn^14^ observed that when confronted with anomalies, scientists tend not to renounce their paradigm but modify their theory in order to eliminate any conflict. If an anomaly persists, he argues, the field will enter a period of crisis and debate and the anomaly will eventually be acknowledged; however, even if the first paradigm has gone badly astray, it will only be declared invalid if an alternate candidate is available to take its place. The failure to translate from animals to humans is certainly a significant anomaly for a field of preclinical translational research. At the same time, an alternate paradigm is emerging, in which preclinical researchers use human-focused (non-animal) in vitro methods (eg, human cells, tissues or organoids) to explore disease processes and test drugs. Here, we explore how preclinical stroke researchers concerned with developing pharmaceutical drugs for acute stroke are attempting to resolve the translational anomaly. In particular, we are interested in the extent to which these researchers are attempting to modify their use of animal models, or are moving towards more human-focused approaches. We explore this question by analysing opinion papers in the field, a novel methodology which to our knowledge is the first of its kind.

## Aims

Our specific research questions are:

1. How have researchers using animal models of stroke responded to the poor ability of these models to translate into clinical benefit?
2. What do researchers attribute the translational problems to and what solutions do they propose?
3. Is there evidence of a move towards human-focused methods as a result of poor translation?

## Methods

### Design

Although we took a systematic approach to literature searching and data extraction, this is not a systematic review because the study involves the synthesis of opinions, not findings. We are neither generating new data by synthesising primary research data nor deriving new conclusions based on research evidence; our study simply provides a synthesis of opinions in the field, permitting insight into the range of views and debates occurring, as well as the direction of travel. Furthermore, the analysis of opinion should not be confused with evidence syntheses that use expert opinion as a *substitute* for findings where qualitative and quantitative evidence is lacking.^15^ Here, we use opinions in their own right, as a means of exploring the discourse around animal models of stroke.

### Search strategies

We searched for opinion papers (eg, commentaries, editorials, viewpoints, special reports) about the use of animal models to develop pharmaceutical drugs for *acute* stroke, with no restrictions by date. Papers had to be written by researchers, either preclinical scientists conducting animal studies or clinical research scientists, working in this field. We excluded papers with a narrow, specific or technical focus, papers that did not focus specifically on stroke, papers about rehabilitation treatments or surgery, primary research, systematic reviews, conference abstracts and letters. We used a combination of hand-searching and simple electronic searches, having previously found this to be an effective strategy for locating elusive literature, such as qualitative studies or theoretical papers.^16 17^ For hand-searching, the first author searched two key journals in the field (Stroke, Translational Stroke Research) and the CAMARADES website (http://www.dcn.ed.ac.uk/camarades/), which includes opinion papers on preclinical stroke research. References of papers selected for full screening were scanned for further eligible papers. Papers found serendipitously were also included. The hand-searches identified 57 eligible papers (table 1), the full texts of which were screened by the second author to check eligibility.

**Table 1:** Hand-searches

Hand-searches	Number of eligible papers identified
*Stroke* journal	30
Reference checking	12
*Translational Stroke Research* journal	7
Serendipity	4
CAMARADES website	2
Google Scholar	2
Total	57

Electronic searches were conducted using MEDLINE, Embase (table 2) and Web of Science in July 2018. The Web of Science search was as follows: TOPIC: (stroke OR cerebrovascular disease) AND TOPIC:(animal model* OR stroke model* OR experimental OR preclinical) AND TOPIC:(translation). DOCUMENT TYPES: (LETTER OR REVIEW OR EDITORIAL MATERIAL).

**Table 2:** MEDLINE and Embase searches

Number	Searches	MEDLINE (16 July 2018)	Embase (20 July 2018)
1	Stroke/	84 404	125 226
2	Cerebrovascular Disorders/	45 250	25 542
3	models, animal/	38 525	1 074 611
4	Drug evaluation, Preclinical/	46 548	13 287
5	stroke model.mp.	1317	2067
6	experimental.mp.	1 200 603	1 180 327
7	translation.mp	114 811	153 494
8	preclinical.mp.	118 391	121 118
9	1 or 2	128 496	146 835
10	3 or 4 or 5 or 6 or 8	1 335 115	2 170 750
11	7 and 9 and 10	119	128

Four hundred and thirty-three papers were identified as being potentially relevant. The titles and abstracts of these 433 papers were screened independently by the two authors (80% agreement). Three hundred and twenty-nine papers were initially excluded and the remaining 104 were examined in greater detail. Duplicates were removed as each reviewer developed their shortlist. After discussion between the two reviewers, 30 were selected as potentially relevant, for which full publications were obtained and screened independently by the same two reviewers (77% agreement). Differences were resolved through discussion. On the final day of searching (19 September 2018), the electronic searches were rerun and a further search, restricted to 2018, was conducted using Google Scholar to check for very recent papers.

### Data extraction

The papers were analysed chronologically and data relevant to the three research questions were extracted. Data were first extracted from the three earliest papers and a preliminary data extraction sheet was developed. As data were extracted from subsequent papers, the data extraction sheet was refined, expanded and agreed by the two authors. During this process, a further two papers were excluded: one was ineligible and the other was almost identical to another paper written by the same researcher.

### Data analysis

The data were analysed qualitatively and descriptively. For the most part, we adopted a deductive approach whereby the analysis was guided by the research question. For example, a recommendation for improving translation was, “In preclinical stroke research animals with comorbidities, mixed gender, advanced age, etc. should play an important role to model the complexities of risk factors, patient profiles, and clinical situation as much as possible”. This was coded as “increase representativeness of animal samples” and organised within the larger category of “improve external validity”. However, researchers’ general observations and comments were also examined, allowing other themes to emerge from the data (inductive approach).

## Results

### Searches

**Figure 1:**
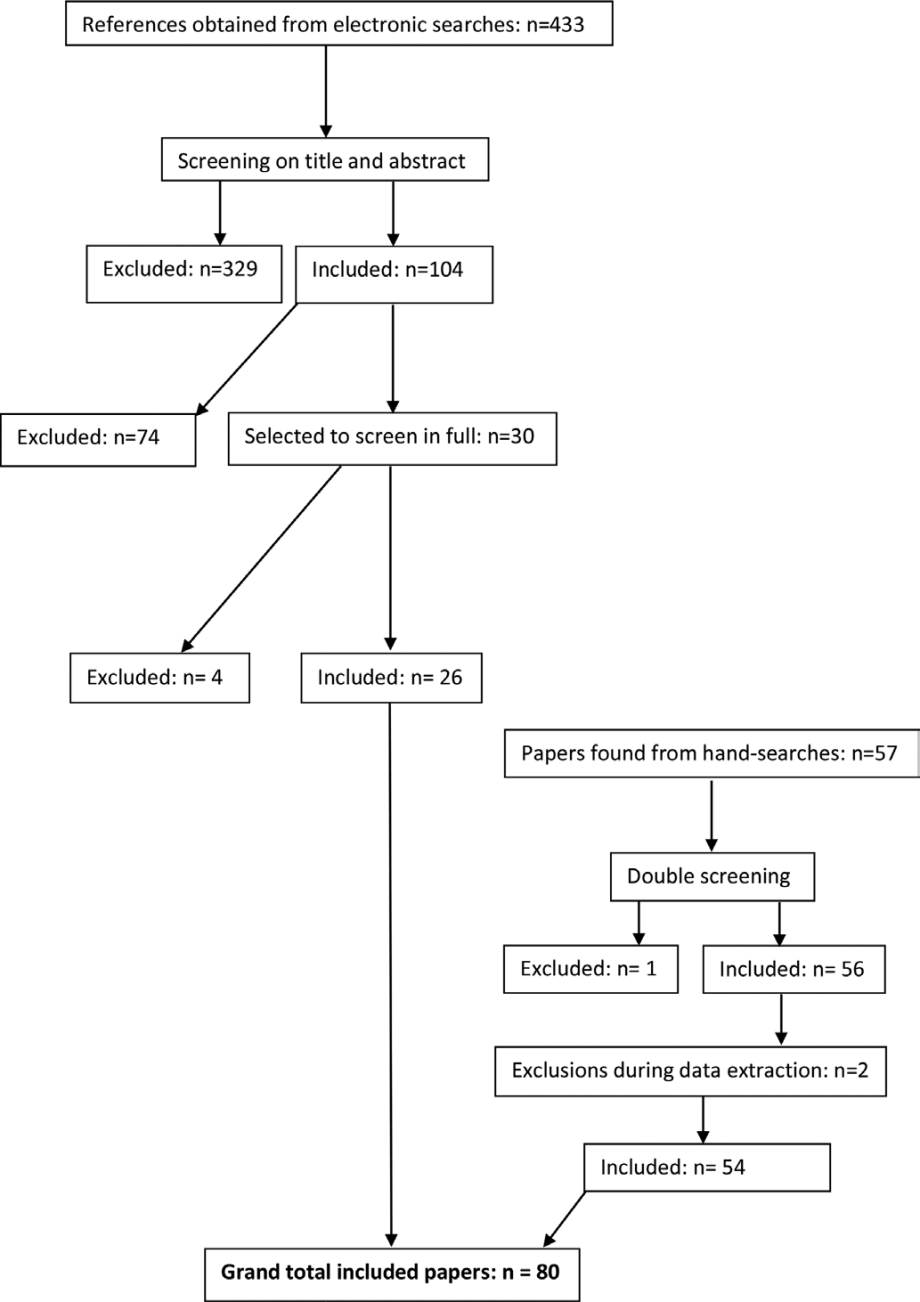
Flow chart showing searches.

Eighty eligible papers were identified, 26 through electronic searches and 54 through hand-searches (figure 1). A list of the papers is provided in the associated dataset https://doi.org/10.5061/dryad.xpnvx0kb9.

### Characteristics of authors and papers

Most authors were located in academic departments of neurology, neuroscience or stroke research (n=62). Seven were in general academic medical research institutions, eight were in a variety of other academic departments and three in pharmaceutical companies (table 3). They were mainly located in the USA (n=36), Germany (n=13), UK (n=10), Australia (n=8) and Canada (n=7), and all were involved in some capacity with the translation of preclinical stroke research. The papers were published between 1979 and 2018. The majority (n=47) discussed issues relating to animal models of stroke in general, while 31 focused on animal models of neuroprotective agents. Most papers discussed ischaemic stroke, but two (both published in 2018) considered animal models of haemorrhagic stroke.

**Table 3:** Author information

	Authors	First author’s country	First author’s main institution	Department
1	Waltz 1979^72^	USA	Dept of Neurology, Pacific Medical Center, San Francisco, CA	Neuro/stroke
2	Molinari 1988^20^	USA	George Washington University Medical Center, 2150 Pennsylvania Avenue NW, Washington, DC	General
3	Wiebers *et al* 1990^18^	USA	Dept of Neurology and Dept of Health Sciences Research, Mayo Clinic and Mayo Foundation, Rochester, Minnesota	Neuro/stroke
4	Zivin and Grotta 1990	USA	Dept of Neurosciences, University of California, San Diego	Neuro/stroke
5	Millikan 1992^21^	USA	Dept of Neurology, Henry Ford Health Sciences Center, Detroit, Michigan	Neuro/stroke
6	Hsu 1993^19^	USA	Division of Restorative Neurology and Human Neurobiology, Baylor College of Medicine, Houston, Texas	Neuro/stroke
7	Hunter *et al* 1995^43^	UK	Smith Kline Beecham Pharmaceuticals, The Pinnacles, Coldharbour Road, Harlow	Pharmaceutical company
8	del Zoppo 1995	USA	Dept of Molecular and Experimental Medicine, Scripps Research Institute, La Jolla, California	Molecular/experimental
9	Grotta 1995	USA	Dept of Neurology, University of Texas Medical School at Houston, Texas	Neuro/stroke
10	Ginsberg 1996	USA	Cerebral Vascular Disease Research Center, Dept of Neurology, University of Miami School of Medicine, Miami, Florida	Neuro/stroke
11	del Zoppo 1998^60^	USA	Dept of Molecular and Experimental Medicine, The Scripps Research Institute, La Jolla, California	Molecular/experimental
12	STAIR 1999^42^	USA	UMass/Memorial Healthcare, 119 Belmont St, Worcester, Massachusetts	General
13	Grotta 1999	USA	University of Texas–Houston Medical School, 6431 Fannin, Houston, Texas	General
14	DeGraba and Pettigrew 2000^69^	USA	Stroke Branch, National Institute of Neurologic Disorders and Stroke, National Institutes of Health, Bethesda, Maryland	Neuro/stroke
15	Gladstone *et al* 2002^64^	Canada	Cognitive Neurology and Stroke Research Unit, Sunnybrook, Toronto, Ontario	Neuro/stroke
16	Grotta 2002	USA	Dept of Neurology, University of Texas–Houston Medical School, 6431 Fannin Street, Houston, Texas	Neuro/stroke
17	Lees 2002	UK	University of Glasgow, University Dept of Medicine and Therapeutics, Western Infirmary, Glasgow	General
18	Davis and Donnan 2002^74^	Australia	Dept of Neurology, Royal Melbourne Hospital, Parkville, Victoria	Neuro/stroke
19	Curry 2003	USA	Dept of Pharmacology and Physiology, University of Rochester, and Stephen H. Curry Consulting, Rochester, New York	Pharmacology/physiology
20	Green *et al* 2003	UK	AstraZeneca R&D Charnwood, Loughborough	Pharmaceutical company
21	Hoyte *et al* 2004^61^	Canada	Calgary Stroke Program, Dept of Clinical Neurosciences, University of Calgary, Calgary, Alberta	Neuro/stroke
22	Cheng *et al* 2004^71^	USA	Stroke Center, University of California School of Medicine, and University of California San Diego, La Jolla, California	Neuro/stroke
23	Fisher and Tatlisumak 2005	USA	Dept of Neurology, University of Massachusetts Medical School, Worcester, Massachusetts	Neuro/stroke
24	Kaste 2005^22^	Finland	Dept of Neurology, Helsinki University Central Hospital, University of Helsinki, Helsinki	Neuro/stroke
25	Donnan and Davis 2005^23^	Australia	National Stroke Research Institute, Austin and Repatriation Medical Centre and University of Melbourne, Melbourne	Neuro/stroke
26	Dirnagl 2006	Germany	Dept of Experimental Neurology, Center for Stroke Research, Humboldt-Universitat Berlin, Universitatsklinikum Charité, Berlin	Neuro/stroke
27	Sena *et al* 2007^44^	UK	Dept of Clinical Neurosciences, University of Edinburgh, Edinburgh	Neuro/stroke
28	Perez de la Ossa and Davalos 2007^24^	Spain	Stroke Unit, Dept of Neurosciences, Hospital Universitari Germans Trias i Pujol, Barcelona	Neuro/stroke
29	Savitz and Fisher 2007^25^	USA	Dept of Neurology, Beth Israel Deaconess Medical Center, Harvard Medical School, Boston	Neuro/stroke
30	Green 2008^53^	UK	Institute of Neuroscience, School of Biomedical Sciences, Queen’s Medical Centre, University of Nottingham, Nottingham	Neuro/stroke
31	Donnan and Davis 2008^75^	Australia	Royal Melbourne Hospital, University of Melbourne, Parkville, Victoria	General
32	Röther 2008^26^	Germany	Dept of Neurology, Klinikum Minden, Academic Teaching Hospital, Hannover Medical School, Minden	Neuro/stroke
33	Hussain and Shuaib 2008^45^	Canada	Division of Neurology, Dept of Medicine, University of Alberta, Edmonton	Neuro/stroke
34	Donnan 2008	Australia	National Stroke Research Institute, Austin Health, University of Melbourne, Waterdale Road, Heidelberg Heights	Neuro/stroke
35	Endres *et al* 2008	Germany	Depts of Neurology and Experimental Neurology, Center for Stroke Research, Charité, Berlin	Neuro/stroke
36	Dirnagl and Macleod 2009^27^	Germany	Depts of Neurology and Experimental Neurology, Charité University Medicine, Berlin	Neuro/stroke
37	Ginsberg 2009^54^	USA	Dept of Neurology, University of Miami Miller School of Medicine, Miami, Florida	Neuro/stroke
38	Fisher *et al* 2009^46^	USA	Dept of Neurology, University of Massachusetts Medical School, Worcester, Massachusetts	Neuro/stroke
39	Bath *et al* 2009^47^	UK	Institute of Neuroscience, School of Biomedical Sciences, Queen’s Medical Centre, University of Nottingham, Nottingham	Neuro/stroke
40	Fisher *et al* 2009^46^	USA	Discovery Translational Medicine, Wyeth Research, 500 Arcola Rd, Collegeville, Pennsylvania	Pharmaceutical company
41	Macleod *et al* 2009^47^	UK	Centre for Clinical Brain Sciences, University of Edinburgh, Edinburgh	Neuro/stroke
42	Tymianski 2010^48^	Canada	Toronto Western Hospital Research Institute, Toronto, Ontario	General
43	Moskowitz 2010	USA	Neuroscience Center, Radiology and Neurology, Massachusetts General Hospital, Harvard Medical School, Boston, Massachusetts	Neuro/stroke
44	Ginsberg 2010	USA	Dept of Neurology, University of Miami Miller School of Medicine, Miami, Florida	Neuro/stroke
45	Turner *et al* 2011^49^	USA	Dept of Neurology, University of California at Davis, Sacramento, California	Neuro/stroke
46	Fisher 2011	USA	Dept of Neurology, UMASS/Memorial Healthcare, Worcester, Massachusetts	Neuro/stroke
47	Budincevic *et al* 2011^55^	Croatia	Dept of Neurology, University Hospital ‘Sveti Duh’, Sveti Duh 64, Zagreb	Neuro/stroke
48	Cook and Tymianski 2011	Canada	University of Toronto, Dept of Surgery, Toronto Western Research Institute Neuroprotection Laboratory, Toronto, Ontario	Neuro/stroke
49	Antonic *et al* 2012^59^	Australia	The National Stroke Research Institute, Florey Neuroscience Institutes, Heidelberg, Victoria	Neuro/stroke
50	Sutherland *et al* 2012^57^	UK	Acute Stroke Programme, Nuffield Dept of Clinical Medicine, University of Oxford, Oxford	Neuro/stroke
51	Lyden and Lapchak 2012^31^	USA	Dept of Neurology, Cedars-Sinai Medical Center, Los Angeles, California	Neuro/stroke
52	Minnerup *et al* 2012^56^	Germany	Dept of Neurology, University of Münster, Albert-Schweitzer-Campus 1 to 48 149 Münster	Neuro/stroke
53	Howells *et al* 2012^29^	Australia	Florey Neuroscience Institutes, Melbourne Brain Centre, Heidelberg, Victoria	Neuro/stroke
54	Dirnagl and Fisher 2012^28^	Germany	Center for Stroke Research, Charité Universitätsmedizin, Berlin	Neuro/stroke
55	Dirnagl *et al* 2013^65^	USA	Dept of Neurology, University of Massachusetts Medical School, 119 Belmont Street, Worcester, Massachusetts	Neuro/stroke
56	Xu and Pan 2013^66^	China	Dept of Neurology, Nanfang Hospital, Southern Medical University, Guangzhou	Neuro/stroke
57	Greenberg 2013^34^	Canada	Buck Institute for Research on Aging, 8001 Redwood Blvd, Novato, California	Ageing research
58	Howells and Macleod 2013^50^	Australia	Florey Institute of Neuroscience and Mental Health, Heidelberg, Victoria	Neuro/stroke
59	Lapchak 2013^32^	USA	Dept of Neurology, Cedars-Sinai Medical Center, Los Angeles, California	Neuro/stroke
60	Dirnagl *et al* 2013^65^	Germany	Dept of Neurology and Experimental Neurology, Center for Stroke Research, Charité Universitätsmedizin Berlin	Neuro/stroke
61	Herson and Traystman 2014^33^	USA	Dept of Pharmacology, University of Colorado Denver, Anschutz Medical Campus, Aurora, Colorado	Pharmacology
62	Lo 2014	USA	Depts of Neurology and Radiology, Massachusetts General Hospital, Harvard Medical School, Charlestown	Neuro/stroke
63	Dirnagl and Endres 2014^30^	Germany	Depts of Neurology and Experimental Neurology, Center for Stroke Research Berlin, Charité Universitätsmedizin, Berlin	Neuro/stroke
64	Neuhaus *et al* 2014^58^	UK	Acute Stroke Programme, Radcliffe Dept of Medicine, University of Oxford, Oxford	Neuro/stroke
65	Howells *et al* 2014^11^	Australia	Florey Institute of Neuroscience and Mental Health, 245 Burgundy Street, Heidelberg, Victoria	Neuro/stroke
66	Boltze *et al* 2014^35^	Germany	Fraunhofer Institute of Cell Therapy and Immunology, University of Leipzig, Leipzig	Cell therapy
67	Dirnagl 2014	Germany	Depts of Neurology and Experimental Neurology Charité, Center for Stroke Research Berlin, Charité, ExcellenceCluster NeuroCure—Universitätsmedizin Berlin, Berlin	Neuro/stroke
68	Offner 2014^37^	USA	Neuroimmunology Research R&D-31, Veterans Affairs Medical Center, 3710 SW Veterans Hospital Rd, Portland	Neuro/stroke
69	Sharp and Jickling 2014^68^	USA	University of California at Davis, MIND Institute, 2805 50th St, Sacramento, California	Neuro/stroke
70	Dirnagl 2016^38^	Germany	Dept of Experimental Neurology, Center for Stroke Research, Charité Universitätsmedizin Berlin	Neuro/stroke
71	Boltze and Ayata 2016^39^	Germany	Fraunhofer Research Institution for Marine Biotechnology, University of Lübeck, Mönkhofer Weg 239a, 23 562 Lübeck	Marine biotech
72	Boltze and Ayata 2016^39^	Germany	Fraunhofer Institute for Cell Therapy and Immunology, Leipzig	Cell therapy
73	Neuhaus *et al* 2017^40^	UK	Acute Stroke Programme, Radcliffe Dept of Medicine, University of Oxford, Oxford	Neuro/stroke
74	Zerna *et al* 2017^41^	Canada	Calgary Stroke Program, Dept of Clinical Neurosciences, Hotchkiss Brain Institute, University of Calgary, Alberta	Neuro/stroke
75	Bosetti *et al* 2017^76^	USA	National Institute of Neurological Disorders and Stroke, National Institutes of Health	Neuro/stroke
76	Lapchak 2017	USA	Dept of Neurology and Neurosurgery, Cedars-Sinai Medical Center, San Vicente Blvd, Los Angeles, California	Neuro/stroke
77	Marbacher 2017	Switzerland	Kantonsspital Aarau, Aarau, Switzerland	General
78	Suzuki and Nakano 2018^67^	Japan	Dept of Neurosurgery, Mie University Graduate School of Medicine, 2-174 Edobashi, Tsu, Mie 514-8507	Neuro/stroke
79	HEADS 2018^62^	USA	Division of Stroke, Dept of Neurology, Beth Israel Deaconess Medical Center, Boston, Massachusetts	Neuro/stroke
80	Bix *et al* 2018	USA	Center for Advanced Translational Stroke Science, University of Kentucky, Lexington, Kentucky	Neuro/stroke
		USA: 36; Germany: 13; UK: 10; Australia: 8; Canada: 7; Japan, Switzerland, China, Croatia, Spain, Finland: 1 each	Depts of neurology, neurosciences, neurosurgery, cerebrovascular disease, stroke research: 62; General academic medical research depts: 7; Pharmaceutical companies: 3; Molecular/experimental depts: 2; Cell therapy depts: 2; Pharmacology/physiology depts: 2; Marine biotechnology: 1; Ageing dept: 1	

### Research findings

First, we consider how researchers using animal models of stroke have responded to the poor ability of these models to translate to humans. Second, we examine what researchers attribute the translational problems to and what solutions they propose, including their views about whether improvements in the scientific rigour of animal studies would increase clinical translation. Third, we explore whether there is evidence of a move towards human-focused approaches as a result of poor clinical translation.

#### How have researchers using animal models of stroke responded to the poor ability of these models to clinically translate?

Almost 30 years ago, Wiebers *et al*^18^ predicted that overreliance on animal models would impede rather than advance progress in treating stroke, and Hsu,^19^ noting that interventions that worked in animals a decade earlier had failed to translate clinically, suggested this challenged the legitimacy of using animal models for stroke. At the same time, several early commentators warned about the dangers of raising expectations, cautioning that overselling ‘promising’ results would lead to the launch of expensive, but ultimately disappointing, clinical trials,^19 20^ while at the same time increasing scepticism about preclinical stroke research.^21^ Some suggested that advances in stroke medicine had occurred without the use of animal models, and that case selection and patient management in clinical trials had exerted the major impact on patient care.^22 23^

In 2007, the failure of the free-radical scavenger NXY-059 represented a huge setback in translational stroke research.^1^ NXY-059 was shown to be neuroprotective in several preclinical studies using rats and primates and indeed the first clinical trial (SAINT-I) found a reduction of global disability. However, SAINT-II failed to replicate the results of SAINT-I and NXY-059 was withdrawn from further development.^24–26^ Writing about the reasons for this failure, Dirnagl and Macleod^27^ suggested that the most troubling explanation—that animals do not model human stroke with sufficient fidelity to be useful—lacked evidence, but acknowledged that the field had not yet demonstrated proof of concept. Success, however, was not forthcoming and before long pharmaceutical companies started exiting^28^ and the concept of human neuroprotection was in doubt.^29 30^ Drawing parallels with the mythological King Sisyphus (condemned to push a large boulder to the top of a hill, only to have to continually repeat the exercise each time the boulder rolled to the bottom), Lyden and Lapchak noted that the repeated failures within translational stroke research were sapping the energy and enthusiasm of collaborators and funders.^31^ It was also observed that some prominent clinical trials had been halted due to lack of efficacy or significant adverse events.^32^

By 2014, it was acknowledged that widespread doubt now existed about the validity of using animal models as predictive tools in stroke, given the remarkable number of neuroprotective agents that had shown promise in animals but gone on to fail in humans.^33^ Similarly, Greenberg noted that although animal models of stroke had been available for over 50 years, few advances in the clinical treatment of acute stroke had occurred.^34^ The sense of crisis^35^ was deepened by a widely publicised article outside the field that demonstrated poor correlation between human and mouse genomic responses to acute inflammatory insults,^36^ with concern expressed for stroke researchers using mice to understand inflammation in human stroke.^37^

By 2016, it was accepted that bench-to-bedside translation in stroke had a ‘disappointing track record’^38^ and that neuroprotection in particular had been a ‘spectacular’^39^ or ‘notable’^40^ failure. Dirnagl^38^ noted that while stroke incidence, morbidity and mortality had decreased, and stroke units and recanalisation had benefited patients, none had been due to ‘bench-to-bedside’ translation. Zerna *et al*^41^ made a similar point with regard to recanalisation. Observing that more than 90% of patients who had an acute stroke still lacked treatment to limit injury or improve outcome, it was proposed that translational stroke research was now at a turning point^39^ and faced a ‘substantial transition’.^41^

#### What do researchers attribute translational problems to and what solutions do they propose?

**Figure 2:**
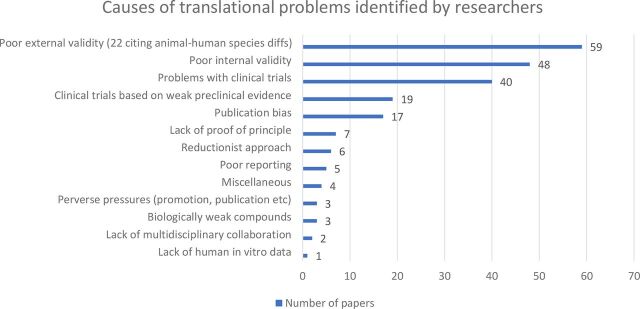
Causes of translational problems identified by researchers.

In terms of researchers’ views on the causes of translational problems, the majority fell into three categories: poor external validity of animal studies (n=59), poor internal validity of animal studies (n=48) and problems relating to clinical trials (n=40). In terms of poor external validity, researchers noted issues such as the inability of animal models to mimic human disease progression, difficulty recapitulating human risk factors, polypharmacy or comorbidity, the use of clinically irrelevant outcome measures and animal–human species differences. In terms of poor internal validity, researchers noted problems such as failure to control bias, lack of pre-trial sample size calculations, inappropriate analyses and poor physiological monitoring. With respect to problems relating to clinical trials, issues such as insufficient selectivity in targeting patients, underpowered trials and late administration of experimental drugs were identified. The associated dataset https://doi.org/10.5061/dryad.xpnvx0kb9 lists the subcategories of the three main explanations identified. Several other explanations for translational problems were identified, including proceeding to clinical trials on the basis of weak preclinical evidence (n-19) and publication bias (n=17). Only a small proportion of the responses cited causes that fundamentally challenged the use of animal models; one cited lack of human in vitro data and 22 within the category of poor external validity cited animal–human species differences (figure 2).

**Figure 3:**
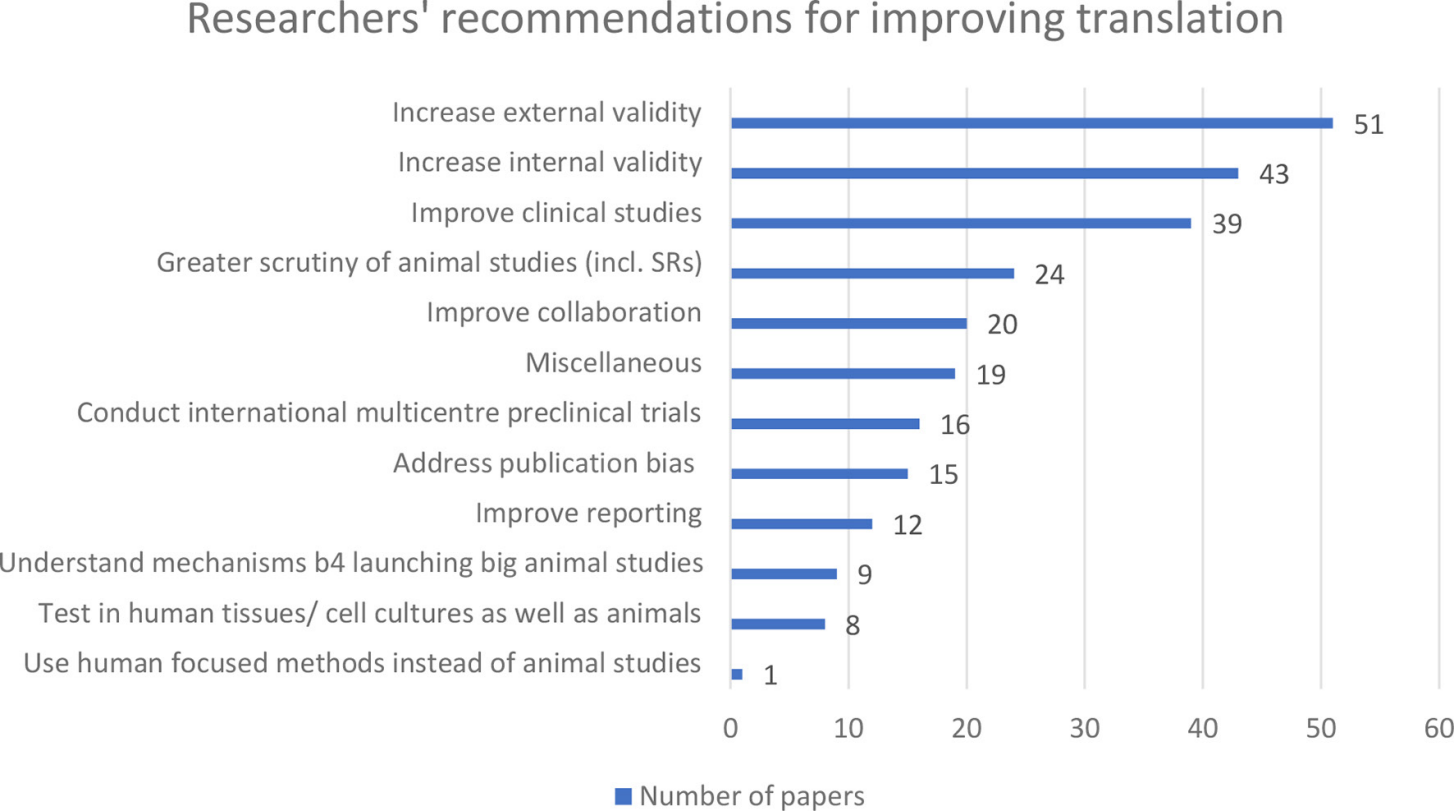
Researchers’ recommendations for improving translation.

In terms of solutions, the most popular recommendation (suggested in 51 papers) was to improve external validity. Included within this category were recommendations such as making animal models more relevant to human stroke, making the timing, dose and route of drug administration more relevant to the human situation, and testing in a variety of animal models and species, including non-human primates (NHPs). The second most popular recommendation (n=43) was to increase internal validity, including taking measures to reduce the risk of bias and adhering to the STAIR guidelines^42^ for improving scientific rigour in preclinical stroke research. Thirty-nine papers recommended that clinical studies should be improved, including suggestions for better patient selection and the earlier administration of experimental drugs. Other proposals included greater scrutiny of animal studies by journal editors, reviewers and those planning clinical trials, together with systematic reviews and audits of animal studies (n=24), greater interdisciplinary collaboration, including between basic and clinical scientists and between academia and pharmaceutical companies (n=20), conducting international multicentre preclinical trials (n=16), tackling publication bias (n=15) and improving reporting (n=12). The associated dataset https://doi.org/10.5061/dryad.xpnvx0kb9 lists the subcategories of the main recommendations given. Again, only a small proportion of recommendations fundamentally challenged the use of animal models; eight papers suggested testing in human tissues/cell cultures as well as animals and one recommended that human-focused methods be used in place of animal studies (figure 3).

While improvements in the internal and external validity of animal studies were popular recommendations, there was disagreement about whether such improvements would increase clinical translation. Many were of the view that they would,^43–49^ with some arguing that the neuroprotection hypothesis, for example, had not yet been refuted because it had not so far been tested in sufficiently rigorous studies.^33 48 50^ There was a reluctance to relinquish the concept of neuroprotection^51^ even after the failure of the SAINT-II trial; the view was that with rigorous translational science, neuroprotection in humans would still be possible.^28 29 50 52^ There was a view that improvements in the scientific quality of preclinical studies, together with ongoing improvements in clinical trial design, would eventually lead to successful translation.^33^ As Dirnagl claimed, translational stroke research was not broken, its engine simply needed overhauling.^38^

Others, however, were more pessimistic. Some suggested that improvements in animal models or study design would not improve clinical translation to a sufficiently high degree,^22 24^ and it was noted that the failure of NXY-059, “*a compound developed with close regard to the best currently established guidelines of preclinical and clinical methodology*”, raised major questions about the value of animal models.^53^ Several pointed out that the STAIR guidelines had failed to improve clinical translation^33 54–57^ and that even perfect implementation of the guidelines might not lead to successful clinical trials^58^ because animal models of stroke might be too dissimilar to human stroke.^33^

#### Is there evidence of a move towards more human-focused approaches?

As noted above, some researchers’ views on the causes of poor translation challenged the animal model paradigm. Antonic *et al*^59^ suggested that poor rates of clinical translation could be due to a lack of human in vitro models and that more human-focused approaches might be necessary to make progress. Furthermore, 22 of the 59 papers that cited problems of external validity as causes of poor translation raised the issue of animal–human species differences, identifying key differences in terms of anatomy, physiology and disease manifestation, pharmacokinetics and treatment response, immune response and genetics (box 1).

Box 1 **Animal–human species differences highlighted by researchers**

**Variations in anatomy, physiology and disease manifestation**

Differences between rodents and humans in vascular anatomy,^18^ collateral blood supply,^52 60^ blood flow and metabolic rate.^55^ Differences in neuroanatomy: rodents have small lissencephalic (smooth) brains with decreased cortical surface area to brain mass compared with humans who have large gyrencephalic brains with multiple folds of the cortical surface.^52 59^ Detailed architecture within cortex is different, with variations in functional maps and synaptic density.^59^ Rodents have relatively little white matter compared with humans.^52 55 59 61 62 71^ Differences between rodents and humans in stroke pathophysiology,^38 63 70^ histopathology,^18^ process of excitotoxicity,^59^ infarct size,^64 72^ location of ischaemic strokes,^68^ recovery from neurological deficits^66^ and possibly mechanisms of brain injury.^63^

**Differences in pharmacokinetics and treatment response**

Species variations in pharmacokinetics, drug dosage, side effects and efficacy,^18 19^ for example, administration of equipotent doses of urokinase or streptokinase mediates different thrombolytic responses in humans, chimpanzees and baboons, with greater differences between primates and non-primates.^60^ Hypoxia tolerance and reaction to neuroprotective agents probably differ between humans and rats.^68^ Some drugs might only work in humans.^59^

**Differences in immune response**

Genetic differences between animals and humans may affect immune responses and outcomes.^69^ Rodents and humans have different inflammatory and immune responses to cerebral ischaemia.^49 60 70^ Most animals used to model stroke (rats, mice, rabbits, sheep, macaques, baboons, rhesus monkeys) have greater percentage of lymphocytes compared with neutrophils in peripheral blood compared with humans, and rodents are more resistant to infections than humans after surgery.^70^

**Genetic differences**

Humans and rodents separated by up to 80 million years of evolution, so significant differences exist between the species.^59 65 67^ Although 90% of the gene order is conserved in mice and humans, and although the proportion of mouse genes without any homologue currently detectable in the human genome appears to be less than 1%, the generation of proteins from these genes differs significantly between the two species, indicating important differences in the way human and rodent cells respond to ischaemic stress.^51^ The expression of genes may vary significantly between rodents and humans despite genetic homology.^68 71^ While humans share 90% of their genome with rodents and have 93% homology with the rhesus macaque, a 10% difference implies that up to 3000 genes may be different and that even those genes with homology may have evolved different biochemistry and function. 59 Cannot assume that humans and rodents share identical molecular targets.^59^

However, while these 22 papers highlighted animal–human species differences—an issue that fundamentally challenges the animal model paradigm—most proposed solutions that involved continuing use of the existing paradigm: develop animal models that replicate human pathophysiology and human stroke more faithfully^37 38 60–65^; test in NHPs^52 66^ or unspecified ‘larger animal models’^62 67^; test in specific animal species^49 68^ or two different animal species^52 61 64 69^; address pharmacokinetics.^19^ One suggested focusing on the similarities^70^ and three did not propose any solution.^55 71 72^

Only 3 of the 22 papers proposed solutions that addressed the paradigm challenge presented by animal–human species differences. Wiebers *et al*,^18^ the only authors to completely dismiss the animal model paradigm (‘paradigm rejecters’), recommended focusing on approaches based on human biology instead of trying to perfect animal models. Two papers proposed what we call ‘paradigm bridging’ solutions (a term first coined by Ritzer^73^), that is, continuing use of animal models but alongside in vitro methods using human tissue.^51 59^ Donnan^51^ proposed that for promising neuroprotectants, it should be a requirement to use human in vitro methods before progressing to human in vivo studies. He observed that well-established in vitro models existed (in which tissue is subjected to hypoxic or ischaemic stress by placing it in a sealed chamber from which oxygen and/or glucose is removed) but noted that these almost universally used cell lines or slices from animals rather than humans. He also proposed using magnetic resonance with diffusion-weighted/perfusion-weighted imaging mismatch to ensure that neuroprotectants cross the blood–brain barrier in humans, as well as positron emission tomography (PET to determine whether the ischaemic penumbra is reached, before embarking on later phase human studies. His roadmap relies heavily on human-focused methods but maintains an initial phase of animal research. Antonic *et al*^59^ recommended using human in vitro methods to test prospective neuroprotective agents, noting that in vitro testing was considerably cheaper than in vivo testing. They proposed that drugs found to be effective in human in vitro systems should then be taken into preclinical animal experiments. In cases where drugs appear to work only in humans, they suggested that (in the absence of whole animal data) biological targeting and effect could be confirmed with PET using tracer quantities of the candidate drug. Antonic *et al* noted that since systemic and central inflammatory processes are a key facet of stroke biology and a source of potential therapeutic targets, an ability to test a range of human tissue (not just neurons) was important. They proposed using human embryonic stem cells, anticipating that as culture systems and tissue engineering evolved, it would be possible to develop increasingly realistic in vitro models of stroke. They emphasised that since humans were the target, there was little logic to using animal cells for in vitro testing, particularly since human cell cultures were available at similar cost.

Alongside Donnan^51^ and Antonic *et al*,^59^ six other papers proposed ‘paradigm bridging’ solutions, that is, proposed that tests should be performed in human tissues/cell cultures as well as in animal models, as a means of improving translation. Davis and Donnan^74^ and Donnan and Davis^75^ argued that proof-of-concept studies should be conducted in humans as a prelude to pivotal clinical trials, while Howells *et al*^29^ noted that testing drugs in vitro before embarking on in vivo research was an affordable technique that might enable a quarter of drugs to be excluded from further development. Neuhaus *et al*^40^ stated that in vitro models of stroke offered valuable mechanistic insight into potential neuroprotective candidates, were able to demonstrate the impact of oxygen and glucose deprivation on specific cell types, and provided valuable ‘target validation’ at the molecular level, while enabling more accurate deductions about causality as a consequence of being able to regulate conditions. By 2017, Bosetti *et al*^76^ anticipated that given the increased availability of human cell lines, human tissues, human organoids, induced pluripotent stem cell (iPSC) technologies and high-throughput assays, in vitro strategies (in combination with animal model data) would be increasingly prominent in future drug development strategies. Likewise, the HEADS consortium^62^ noted that emerging human-focused techniques (including primary adult microglial and astrocyte cultures, iPSCs, single-cell isolation from brain slices, and cell phenotyping after flow cytometry) could also be applied in intracerebral haemorrhage research.

## Is paradigm change likely?

**Figure 4:**
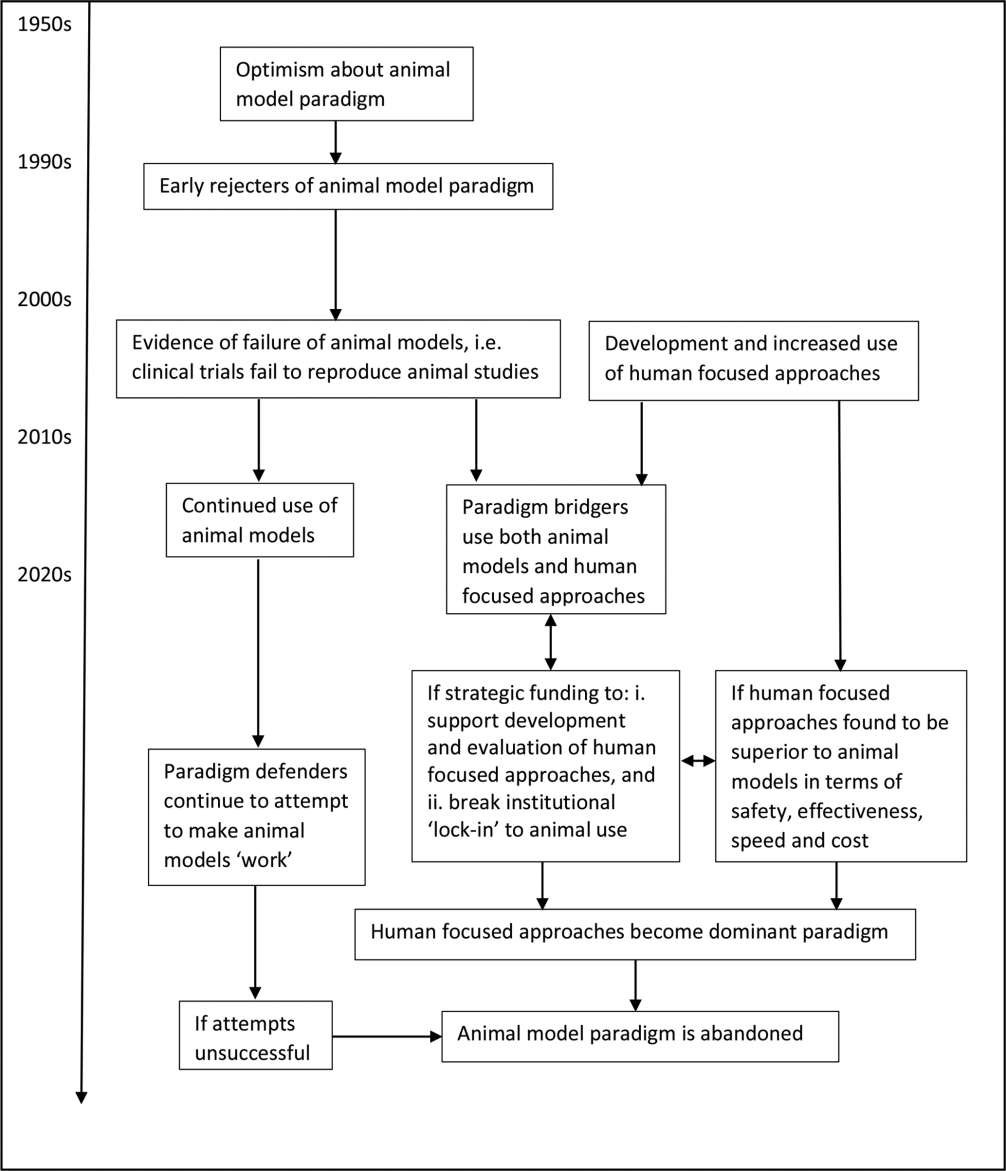
Paradigm change within translational stroke research.

With the increasing development of human-focused approaches and technologies in the field of stroke, alongside accumulating evidence of the translational failure of animal models, it seems likely that the proportion of paradigm bridgers will gradually increase. If human-focused approaches are found to be a safe and effective way forward in drug discovery and testing, and if they offer benefits in terms of speed and cost, they may eventually dominate translational stroke research. However, it seems probable that ‘paradigm defenders’ will continue for some time to try to make the animal model paradigm ‘work’ (figure 4).

## Discussion

The systematic analysis of opinion papers is a novel methodology, and as we have shown, it can provide insight into the state of play within an academic discipline, including the range of views, the sorts of debates occurring and the direction in which the field appears to be heading. However, we recognise that papers published in established journals may not capture the full diversity of opinion within a field since they are likely to be biased towards ‘experts’ and possibly towards those with more conservative views. Nevertheless, if published opinions are more likely to be ‘establishment’ views, these probably give an accurate indication of where the field is heading and how soon it is likely to get there. Locating opinion papers can be challenging since they are not always easily identifiable and in this respect hand-searching played an important role. We feel confident that as a result of combining electronic and hand-searches, we did not miss any relevant papers; towards the end of the search process, we seemed to reach ‘saturation’ level, that is, we repeatedly came across the same papers without identifying any new ones.

We found universal agreement that translational stroke research was in crisis, as well as some robust questioning of animal models. At the same time, however, when it came to identifying the specific causes of this crisis, most researchers’ explanations did not fundamentally challenge the use of animal models, although some cited animal–human species differences and one cited a lack of human in vitro models. Similarly, most of the solutions proposed by researchers—even by those who had highlighted the problem of animal–human species differences—involved fine-tuning aspects of the existing paradigm (although there was some disagreement about whether such modifications would improve clinical translation). A small number of researchers proposed using human in vitro methods alongside animal models and one proposed using only human-focused methods. Although most advocated continued use of animal models then, there is evidence of an emerging challenge in the form of human-focused approaches.

As Kuhn^14^ noted, when confronted with anomalies within a paradigm, scientists tend not to renounce that paradigm but attempt to modify their theory instead. This may explain researchers’ dogged persistence with animal models despite decades of failure. Writing about the tenacity of systems of opinion within science, Fleck^77^ notes that contradictions may appear unthinkable and are often actively resisted, or remain ‘unseen’. Such contradictions within science have been referred to elsewhere as ‘uncomfortable knowledge’.^78^ Kuhn also observed that scientists tend to select problems that can be solved with theories, concepts and instruments close to those already in existence, rather than pursuing new phenomena or theories.^14^ This may help explain why only a minority of researchers in this study had so far begun to explore human-focused methods.

Researchers may also be reluctant to relinquish animal models due to ‘psychological lock-in’, the phenomenon of beliefs persevering in the face of contradictory evidence.^79^ As Frank suggests, for researchers using animal models, belief in the value of their work may have hardened as a result of being questioned about it on ethical grounds. In addition, because of the closed nature of animal research, scientists using animal models may not have been exposed to the usual diversity of academic opinion and debate, leading to beliefs and practices becoming entrenched. Furthermore, they may not perceive it to be in their interests to change; referring to ‘institutional lock-in’, Frank notes that a huge infrastructure perpetuates animal research within universities, whereby academic departments benefit from funding, professional associations, conferences and academic programmes devoted to animal research, all of which make it harder to embrace new approaches.^79^

Nevertheless, it seems appropriate that translational researchers consider new approaches. Animal models have been unsuccessful in the field of stroke and also in the fields of traumatic brain injury,^80^ motor neuron disease,^81^ inflammation,^36^ sepsis,^82^ central nervous system diseases,^83^ Alzheimer’s disease,^84 85^ arthritis,^86^ asthma,^87^ cancer,^88^ multiple sclerosis,^89^ myocardial infarction,^90^ Parkinson’s disease,^90^ type 1 diabetes^91^ and elsewhere,^92^ strongly suggesting that human-focused approaches might have relevance. A range of in vitro techniques has emerged and methods of culture and tissue engineering are continually evolving. With the increased availability of human cells and the development of new technologies such as microfluidic devices, it has been suggested that in vitro systems may improve the efficiency of clinical drug development and reduce drug attrition rates and their associated costs.^93 94^ The Medical Research Council has recently stated that it aims to fund exploration of emerging technologies such as 3D tissue models and organoids.^95^ Researchers using such technologies need to ensure that they attend to issues of internal and external validity, reporting and publication bias,^96^ otherwise they risk making the same mistakes as those conducting animal studies. Furthermore, even though the animal model paradigm has gone badly astray, Kuhn’s^14^ theory suggests that it will only be declared invalid if an alternate candidate is available to take its place—and an alternate candidate will only be seriously considered if it is valid, reliable and fit for purpose. Consequently, validity and reliability are of paramount importance in the development of new, human-focused approaches. The speed of any transition away from animal models will depend on the extent to which funding is redistributed, both to explore the evidence for new approaches and to break lock-in to the use of animal models. There is an urgent need to revitalise translational stroke research and pursue fresh approaches because the economic burdens of stroke are enormous^97^ and also because the personal costs for patients who had a stroke are huge.^98–100^

## Conclusions

Despite frank acknowledgement that animal models have not been fruitful in the field of stroke, most researchers in the field exhibited a strong resistance to relinquishing this type of research. Nevertheless, there is evidence of an emerging challenge to the use of animal models, in the form of human-focused in vitro approaches. For the sake of stroke patients, whose needs have been unmet for so long, there is a pressing requirement to investigate the validity of these new approaches.
